# Targeting EZH2 Promotes Chemosensitivity of BCL-2 Inhibitor through Suppressing PI3K and c-KIT Signaling in Acute Myeloid Leukemia

**DOI:** 10.3390/ijms231911393

**Published:** 2022-09-27

**Authors:** Chan Yang, Yan Gu, Zheng Ge, Chunhua Song

**Affiliations:** 1 Department of Hematology, Zhongda Hospital, School of Medicine, Southeast University, Institute of Hematology Southeast University, Nanjing 210009, China; 2 Hershey Medical Center, Pennsylvania State University Medical College, Hershey, PA 17033, USA; 3 Division of Hematology, The Ohio State University Wexner Medical Center, The James Cancer Hospital, Columbus, OH 43210, USA

**Keywords:** EZH2 inhibitor, BCL-2 inhibitor, acute myeloid leukemia, PIK3IP1, c-KIT

## Abstract

Acute myeloid leukemia (AML) is one of the most common hematological malignancies with high heterogeneity, characterized by a differentiating block at the early progenitor stage. The selective BCL-2 inhibitor, Venetoclax (Ven), has shown exciting clinical results in a certain group of AML patients. However, Ven alone is insufficient to reach an enduringly complete response, which leads to the concern of Ven resistance. Alternative combined therapies with Ven are demanded in AML. Here, we reported the synergistic effect and molecular mechanism of the enhancer of zeste homolog 2 (EZH2) inhibitor DZNeP with Ven in AML cells. Results showed that the combination of DZNeP with Ven significantly induces cell proliferation arrest compared to single-drug control in AML cells and primary samples, and CalcuSyn analysis showed their significant synergy. The combination also significantly promotes apoptosis and increases the expression of pro-apoptotic proteins. The whole transcriptome analysis showed that phosphoinositide-3-kinase-interacting protein1 (PIK3IP1), the PI3K/AKT/mTOR signaling suppressor, is upregulated upon DZNeP treatment. Moreover, EZH2 is upregulated but PIK3IP1 is downregulated in 88 newly diagnosed AML cohorts compared to 70 healthy controls, and a higher expression of EZH2 is associated with poor outcomes in AML patients. Particularly, the combination of DZNeP with Ven dramatically eliminated CD117 (c-KIT) (+) AML blasts, suggesting the effect of the combination on tumor stem cells. In summary, our data indicated that DZNeP increases the sensitivity of Ven in AML by affecting PI3K and c-KIT signaling in AML. Our results also suggested that the therapeutic targeting of both EZH2 and BCL-2 provides a novel potential combined strategy against AML.

## 1. Introduction

Acute myeloid leukemia (AML) is the most common acute leukemia in adults, accounting for ~80 percent of cases in this group [1]. Despite advancements in therapeutic regimens in recent decades, the poor clinical outcome and extensively high relapse rate in the elderly population remain a challenge [2,3].

The antiapoptotic protein, B-cell lymphoma 2 (BCL-2), is overexpressed in AML, specifically in leukemic stem cells (LSC), and its dysregulation is related to chemotherapy resistance and poor clinical outcomes [4,5,6,7]. Venetoclax (Ven, ABT-199) is a BH3 mimetic and a BCL-2 selective inhibitor [8,9]. The FDA approved Ven in combination with hypomethylating agents (HMAs, such as azacitidine and decitabine) or low-dose cytarabine (LDAC) for the treatment of newly diagnosed AML in adults ineligible for intensive chemotherapy in 2018 [10,11,12]. Although the initial response rates of 60–80% of HMA + Ven or LDAC + Ven treatment are highly encouraging in newly diagnosed, elderly, unfit patients, unfortunately, most patients have relapsed after a median duration response of 8–11.3 months [11]. These data indicate that the novel combination of BCL-2 inhibitors with other agents is highly demanded in AML.

The enhancer of zeste homolog 2 (EZH2) is a histone methyltransferase and is the catalytic subunit of the polycomb repressive complex 2 (PRC2) [13,14,15]. EZH2 genomic amplification and overexpression occur across many types of cancers, including AML, and enhance tumorigenesis [16,17,18]. The epigenetic therapy targeting EZH2 has invoked plentiful concern [19,20]. DZNeP (3-deazaneplanocin A) is the representative EZH2 inhibitor that effectively inhibits the activity of S-adenosyl-L-homocysteine hydrolase, thereby restraining the activity of the EZH2 methyltransferase and depressing the trimethylation of the histone 3 lysine 27 [21]. Moreover, DZNeP has been reported to target cancer cells but protect normal cells, while effectively targeting subpopulations of cells with stem-like properties [22,23,24]. The combination of EZH2 inhibitors with other targeted drugs showed a significant therapeutic effect in AML cells [25,26,27]. However, the anti-leukemia activity of DZNeP in combination with BCL-2 inhibitors has not been reported so far.

c-KIT (CD117), known as the classical expression marker of immaturity blasts, is a receptor tyrosine kinase (RTKs) that is expressed on the surface of hematopoietic stem/progenitor cells [28]. Tumor cells positively expressing c-kit exhibited cancer stem cell characteristics, such as self-renewal and chemoresistance [29,30,31]. c-kit is involved in cancer stem cell-mediated progression and also therapeutic resistance [32,33]. However, the role of c-KIT in leukemia stem cells (LSCs) and the BCL-2 inhibitor resistance is not well explored.

In this study, we sought to investigate the synergistic effect and molecular mechanism of the combined EZH2 inhibitor, DZNeP, with the BCL-2 inhibitor, Ven, in AML cells. Our results support that the concomitant treatment of DZNeP and Ven causes more inhibition of proliferation and induces synergistic cell death in AML compared to monotherapies not only in cell lines, but also in AML primary blasts with the expansion of c-KIT, which suggests that c-KIT may be recognized as a specific biomarker which has a promising response to this dual-suppressed BCL-2 and EZH2 blockade by Ven and DZNeP.

## 2. Results

### 2.1. DZNeP Potentiates the Proliferation Inhibition of Ven in AML Cells

**Figure 1:**
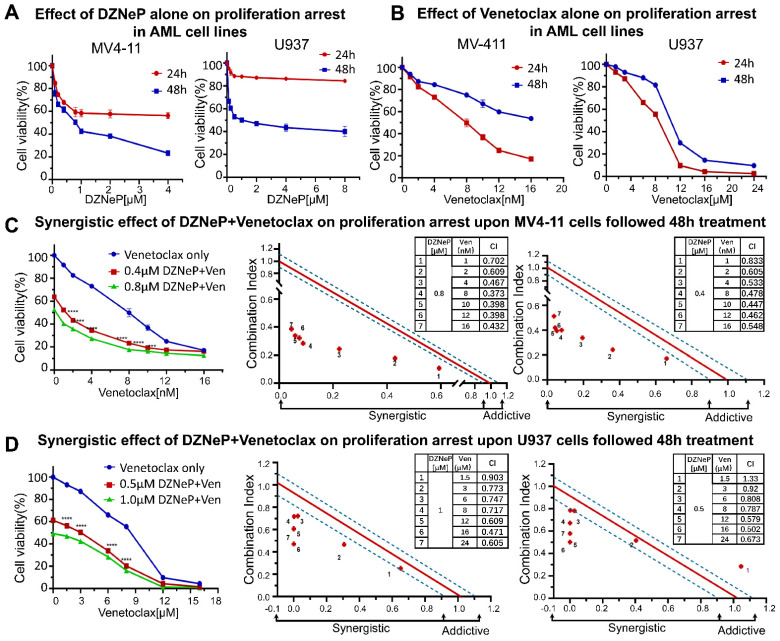
Synergistic effects of DZNeP with Ven on cell proliferation arrest in AML cell lines. (**A**,**B**) Effects of DZNeP or Ven alone on the proliferation of MV4-11 and U937 cells. Cells were treated with the indicated doses of drugs for 24 and 48 hrs. Cellular viability was measured by a CCK-8 assay. (**C**,**D**) Synergistic effects of Ven in combination with 0, ½IC50, or IC50 doses of DZNeP on the proliferation of MV4-11 and U937 cells. Synergistic analysis was performed with Calcusyn, where a combination index value of 1.1 to 0.9 is considered an additive effect, <0.9 is a synergistic effect, and >1.1 is an antagonistic effect, respectively. Mean ± SD of triplicates is representative of 1 of 3 independent experiments. ***, *p* < 0.001. **** *p* <0.0001.

The effects of DZNeP or Ven alone on cell proliferation arrest were first explored in AML cell lines by the CCK-8 assays. Results showed that DZNeP displayed a modest inhibitory effect on the cell viability of MV4-11 and U937 AML cells in both a dose-dependent and time-dependent manner (Figure 1A), whereas Ven markedly increased the inhibition rate of cell viability in a much more significantly dose-dependent manner (Figure 1B). The IC50 of DZNeP was 0.73 μM upon MV4-11 and 1.28 μM upon U937 cells after being treated for 48 h. Compared with U937 cells (IC50 = 7.62 μM), MV4-11 cells were much more sensitive to Ven (IC50 = 6.88 nM), in which IC50 was almost 1000 times smaller than that in U937 cells. These results indicated that U937 is a relatively resistant cell line to Ven, while MV4-11 is the sensitive one, which is consistent with the previous report [34].

Next, we examined the synergistic effect of DZNeP with Ven for 48 h (Figure 1C,D). Results showed that DZNeP at a concentration of half-dose IC50 or IC50 significantly enhanced the Ven-mediated inhibition of cell proliferation in not only the sensitive cell line, MV4-11, but also the resistant U937 cells compared to single-drug controls. CalcuSyn analysis showed the synergistic effect of the DZNeP + Ven on cell proliferation arrest.

### 2.2. Synergistic Effect of DZNeP with Ven on Apoptosis of AML Cells

**Figure 2:**
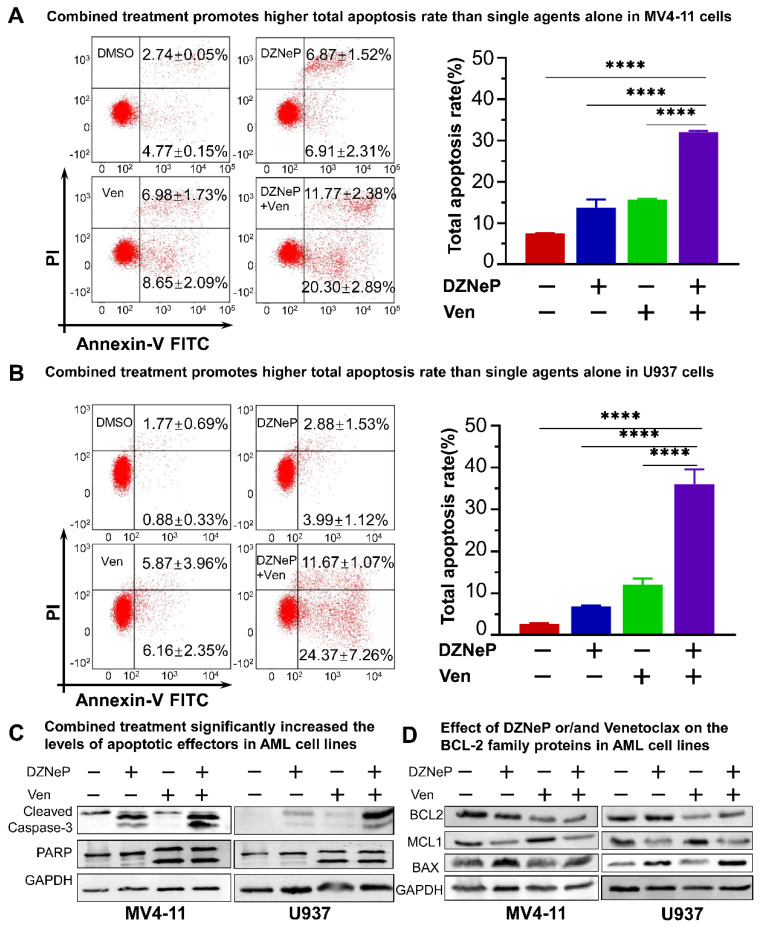
Synergistic effects of DZNeP with Ven on apoptosis in AML cells. (**A**,**B**) Effects of DZNeP (1 μM), Ven (8 nM for MV4-11 and 8 μM for U937, respectively), and the combination of DZNeP (1 μM) plus Ven (8 nM for MV4-11 and 8 μM for U937, respectively) on apoptosis in MV4-11 (**A**) and U937 cells (**B**). Cells were treated for 48 hrs and were stained with PI and annexin V FITC for flow cytometry analysis. The percentage of the total apoptosis rate is calculated with the early apoptotic plus the late apoptotic proportion. Mean ± SD of triplicates is representative of independent experiments. ****, *p* < 0.0001. (**C**) Western blot analysis of the apoptotic effectors, cleaved caspase-3 and PARP, in MV4-11 and U937 cells following the treatment of the indicated drugs for 48 hrs. GAPDH was used as a loading control. (**D**) Western blot showing the expression of the BCL-2 family members (BCL-2, MCL1, and BAX) in MV4-11 and U937 cells treated with the indicated drugs for 48 h. GAPDH was used as a loading control.

We examined the effect of Ven on DZNeP-induced apoptosis in AML cells treated with vehicle, DZNeP, Ven, or a combination (DZNeP + Ven) at the indicated concentrations for 48 h. Results showed that the combination of DZNeP with Ven dramatically increased apoptosis in MV4-11 (32.07% ± 0.55) and U937 (36.03% ± 6.19) cells compared to the control and single drugs (*p* < 0.01 for all cases, Figure 2A,B). Western blots revealed that the protein levels of the apoptotic effectors, cleaved caspase-3 and PARP, were significantly enhanced by the combination compared to single-drug and vehicle control (Figure 2C). BCL-2 family members (BCL-2 and MCL-1) play critical roles in the intrinsic apoptotic pathway. Ven alone or in combination with DZNeP decreased the BCL-2 protein in both cell lines (Figure 2D), while Ven single treatment increased the MCL-1 protein as previously reported consistently [35,36], but the combination with DZNeP completely depleted the increase in MCL-1 in both cell lines. Moreover, DZNeP alone or the combination significantly elevated BAX protein levels (Figure 2D).

### 2.3. Roles of PIK3IP1, the Suppressor of PI3K/AKT/mTOR Signaling, in the Combination-Mediated Anti-Tumor Effect

**Figure 3:**
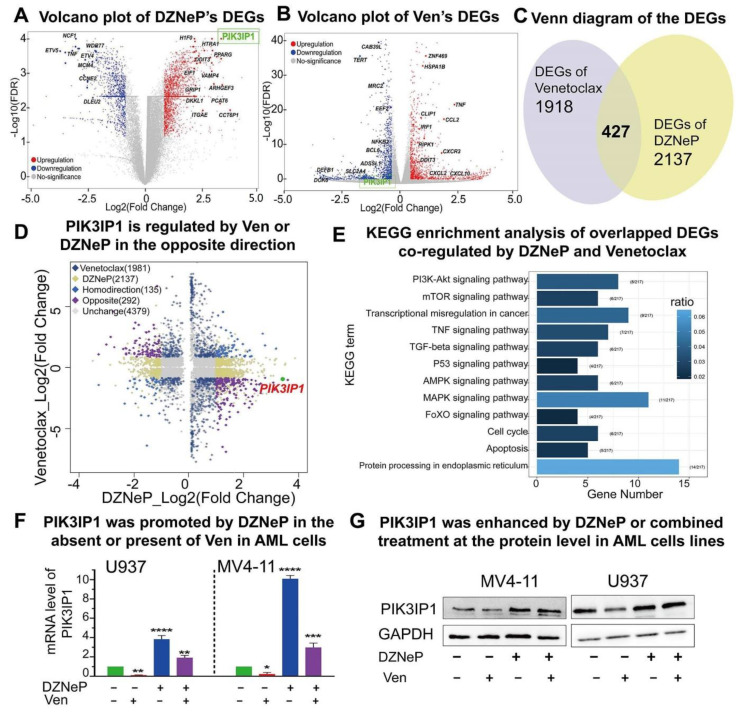
PIK3IP1 plays an important role in the synergistic anti-AML activity upon the combination therapy. (**A**,**B**) RNA-seq identified different expression genes (DEGs) of DZNeP or Ven treatment compared to the vehicle control in U937 cells. RNA-seq was performed with total RNA isolated from U937 cells treated with 2 μM of DZNeP, 7.5 μM of Ven, or vehicle for 48 h. (**C**,**D**) RNA-seq analysis identified 427 overlapped DEGs; 135 genes are regulated in the same direction, and 292 genes are regulated in the opposite, among which PIK3IP1 was induced by DNZeP while inhibited by Ven. (**E**) KEGG enrichment analysis of the overlapped DEGs identified the involved pathways co-regulated by DZNeP and Ven. (**F**) The mRNA expression level of PIK3IP1 induced by DZNeP or/and Ven was measured by qRT-PCR. *, *p* < 0.05, **, *p* < 0.01. ***, *p* < 0.001. ****, *p* < 0.0001. (**G**) The protein expression of PIK3IP1 induced by DZNeP or/and Ven was detected by western blot.

To further understand the mechanisms underlying the synergy of DZNeP with Ven, the whole genome transcriptome was analyzed in U937 cells treated with 2 μM of DZNeP, 7.5 μM of Ven, or vehicle for 48 h, respectively, by RNA-seq. A total of 2345 statistically significant differentially expressed genes (DEGs) were identified in the cells upon Ven treatment compared to the vehicle control, and 2564 DEGs upon DZNeP treatment (Figure 3A,B), in which 427 were overlapped (135 genes changed in the same direction and 292 in the opposite) (Figure 3C,D). KEGG enrichment analysis of the overlapped DEGs showed the significant enrichment of genes in multi-oncogenic signaling (PI3K/AKT/mTOR, MAPK, etc.), the cell cycle progress, and the apoptosis signaling pathway (Figure 3E).

As highlighted in Figure 3A,B, the expression of key genes in PI3K and MAPK and apoptosis signaling are significantly altered upon the drug treatment. Particularly, PIK3IP1, the suppressor of the PI3K/AKT/mTOR signaling pathway, is upregulated upon DZNeP treatment but downregulated upon Ven treatment. Both RT-qPCR and western blot analyses showed that DZNeP treatment alone upregu-lates but Ven alone downregulates the expression of PIK3IP1 (Figure 3F,G). These data suggested that PIK3IP1 is a key gene in the combination of Ven with DZNeP and also revealed that DZNeP enhances the expression of PIK3IP1 to sensitize AML cells to Ven treatment.

### 2.4. PIK3IP1 Dependence on the Synergy of DZNeP with Ven in AML Cells

**Figure 4:**
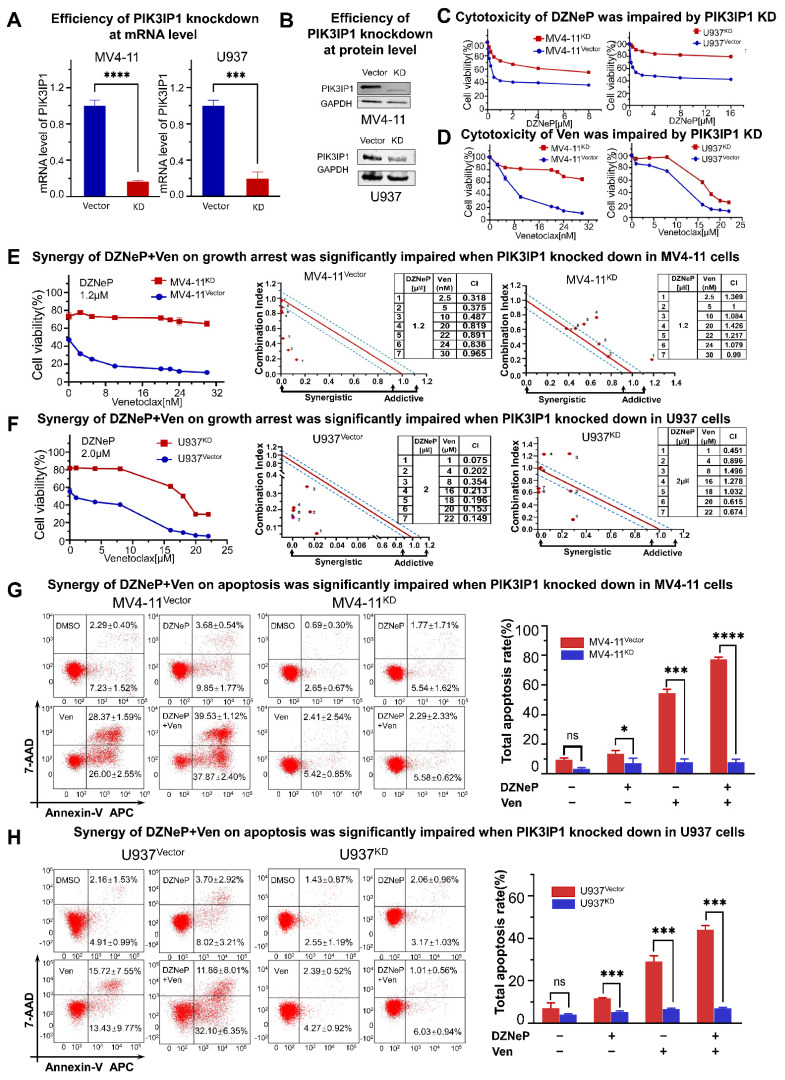
The mechanism of the synergy of co-targeting EZH2 and BCL-2 by DZNeP + Ven is in a PIK3IP1-dependent manner. (**A**) qRT-PCR was used to measure the efficiency of PIK3IP1 knockdown by shRNA in MV4-11 and U937 AML cells. (**B**) Western blot was performed to measure the efficiency of PIK3IP1 knockdown by shRNA in MV4-11 and U937 AML cells. (**C**,**D**) Effects of the single agents upon proliferation arrest were markedly dampened after PIK3IP1 was knocked down in MV4-11 and U937 AML cells. (**E**,**F**) Synergistic effects of the combination treatment upon growth inhibition were significantly impaired when PIK3IP1 was knocked down in MV4-11 and U937 cells. (**G**,**H**) Synergistic effects of the combination treatment upon apoptosis were significantly decreased in PIK3IP1 knockdown cells compared to the vector-transfected cells. ns, no significant. *, *p* < 0.05. ***, *p* < 0.001. **** *p* < 0.0001.

To deeply understand the role of PIK3IP1 in the synergy of DZNeP and Ven, PIK3IP1 was knocked down in U937 and MV4-11 cells. The results of the qPCR and western blots showed that PIK3IP1 expression was effectively reduced in both mRNA and protein levels compared to the scramble shRNA control (shCTL) (Figure 4A,B). PI3KIP1 knockdown significantly increased the tolerance to DZNeP or Ven alone (Figure 4C,D) and blocked the synergy of the DZNeP + Ven combination compared to that in the shCTL cells (Figure 4E,F). Apoptosis analysis demonstrated that PIK3IP1 knockdown almost completely rescues the effect of the combination and single drugs on cell death compared to shCTL in MV4-11 (Figure 4G) and U937 cells (Figure 4H). These results indicated that PIK3IP1 is a key mediator for the synergy of the DZNeP + Ven combination, and DZNeP synergized with Ven to induce cell proliferation arrest and apoptosis in a PI3KIP1-dependent manner.

### 2.5. High Expression of EZH2 Is Associated with Poor Outcomes in AML Patients

**Figure 5:**
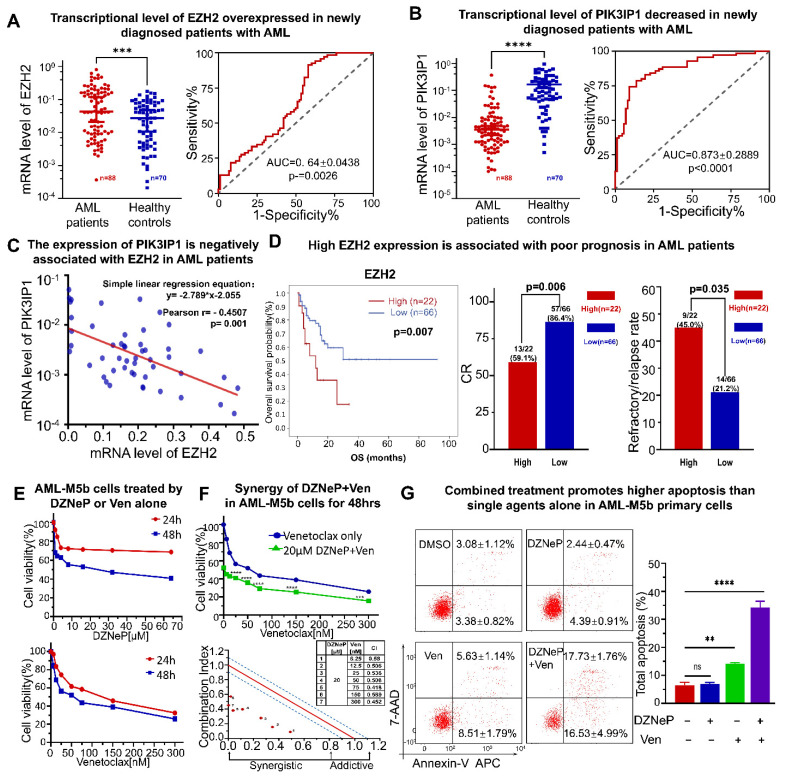
A high expression of EZH2 is associated with poor outcomes in patients with AML and the synergistic effect of DZNeP + Ven in AML-M5b primary blasts. (**A**,**B**) A qRT-PCR assay was performed to measure the expression of EZH2 and PIK3IP1 in newly diagnosed AML patients’ bone marrow samples (*n* = 88) compared to the healthy donors’ PBMC samples (*n* = 70). (**C**) Pearson correlation and a simple linear regression analysis were conducted to show the associated relationship between EZH2 and PIK3IP1. (**D**) Comparison of overall survival (OS), % complete response (CR) rate, and refractory/relapse rate in patients with an EZH2 high expression (high) versus an EZH2 low expression (low) in our AML cohort. (**E**) Effects of DZNeP or Ven single agents on cell growth inhibition in AML-M5b primary blasts. Cells were treated with the indicated doses of drugs for 24 and 48 hrs. Cellular viability was measured by a CCK-8 assay. (**F**) Synergistic effects of Ven in combination with 0 and IC50 doses of DZNeP on cell growth arrest in AML-M5b primary blasts. Synergistic analysis was performed with Calcusyn, where a combination index value of 1.1 to 0.9 is considered an additive effect, <0.9 is a synergistic effect, and >1.1 is an antagonistic effect, respectively. (**G**) Effects of DZNeP (20 μM), Ven (50 nM), and the combination of DZNeP plus Ven on apoptosis in AML-M5b primary cells. Cells were treated for 48 hrs and were stained with hCD34, hCD117 surface biomarkers, the apoptotic antibody 7-AAD, and annexin V APC for flow cytometry analysis. The flow cytometry gating strategy was used for determining the apoptosis rate of the CD34/CD117 dual-positive AML blast subpopulation. The percentage of the total apoptosis rate is calculated with the early apoptotic plus the late apoptotic proportion. Mean ± SD of triplicates is representative of 1 of 3 independent experiments. ns, no significant. **, *p* < 0.01. ***, *p* < 0.001. ****, *p* < 0.0001.

We examined the expression of PIK3IP and EZH2 in newly diagnosed patients with AML and in the healthy controls in our institute. Results displayed that the EZH2 expression is significantly elevated (*p* < 0.0001) (Figure 5A), while PIK3IP1 is concomitantly reduced (*p* < 0.0001) (Figure 5B) in 88 newly diagnosed patients with AML compared to the 70 healthy controls. An EZH2 high expression and a PIK3IP1 low expression were also observed in AML patients compared to the normal controls from the database (Figure S1A,B, left panel). A receiver operating characteristic curve (ROC) was performed, and the corresponding AUC was calculated. The EZH2 expression reached AUC (0.64 ± 0.0438), and the PIK3IP1 reached AUC (0.873 ± 0.2889) (Figure 5A,B). The Pearson correlation analysis found a moderate negative correlation between EZH2 and PIK3IP1 at the mRNA level in our AML cohort (*n* = 50, Pearson r = −0.4507, and *p* = 0.001) (Figure 5C).

We also examined the association of the EZH2 expression with the clinical features of 88 AML patients in our institute. The patients were divided into EZH2-high (*n* = 22) and EZH2-low (*n* = 66) groups based on their mRNA intensity. The clinical features and analyzed results of the patients are shown in Tables S1 and S2. Results show that the overall survival (OS) in the EZH2-high group is significantly shorter than those in the EZH2-low group (*p* = 0.007). The complete response (CR) rate is significantly lower (59.1% vs. 86.4%, *p* = 0.006), but the refractory/relapse rate is higher (45.0% vs. 21.2%, *p* = 0.035) in patients in the EZH2-high group versus those in EZH2-low group (Figure 5D). A lower platelet at diagnosis was observed in the EZH2-high group compared to those in the EZH2-low group (*p* = 0.047). Four AML patients were detected to have a deletion 7 or deletion 7q, a high-risk prognostic factor of AML; three of them are in the group with an EZH2-high expression and one is in the group with an EZH2-low expression (Table S1). No statistical difference was found in gender, FAB subtypes, WBC, Hb, and bone marrow blast cell count between the EZH2-high and EZH2-low groups in our cohort (Table S1). Metadata analysis also showed that AML patients with a high EZH2 expression had a worse outcome (Figure S1B). We also analyzed the association of the PIK3IP1 expression with the clinical features in our cohort. Only significantly higher rates of the genetic defects in the TP53, DNMT3A, and NPM1 genes are observed in the patients with PIK3IP1-low versus PIK3IP1-high expressions (Tables S1 and S2), which also suggests the tumor suppressing role of PIK3IP1 in the disease.

Taken together, these results show that the EZH2 expression is negatively correlated with PIK3IP1 in newly diagnosed AML patients, and an EZH2-high expression is associated with poor outcomes in AML patients.

### 2.6. Synergistic Effect of DZNeP with Ven in c-KIT-Enriched Primary Cells from AML Patients

The primary leukemic cells highly expressing CD34 and c-KIT were obtained from a newly diagnosed patient with the AML-M5b subtype. CCK-8 assay was performed to examine the synergy on cell proliferation of DZNeP with Ven. Results show that the IC50 treated by DZNeP or Ven alone was 19.73 μM and 54 nM, respectively, in the cells for 48 h (Figure 5E), and CalCusyn analysis showed the synergistic effect of DZNeP + Ven on cell growth arrest in the cells (Figure 5F), indicating that DZNeP significantly promotes the effect of Ven. Apoptosis assay also showed a significant effect of the DZNep + Ven combination on the primary cells compared to single-drug controls (*p* < 0.01, Figure 5G).

**Figure 6:**
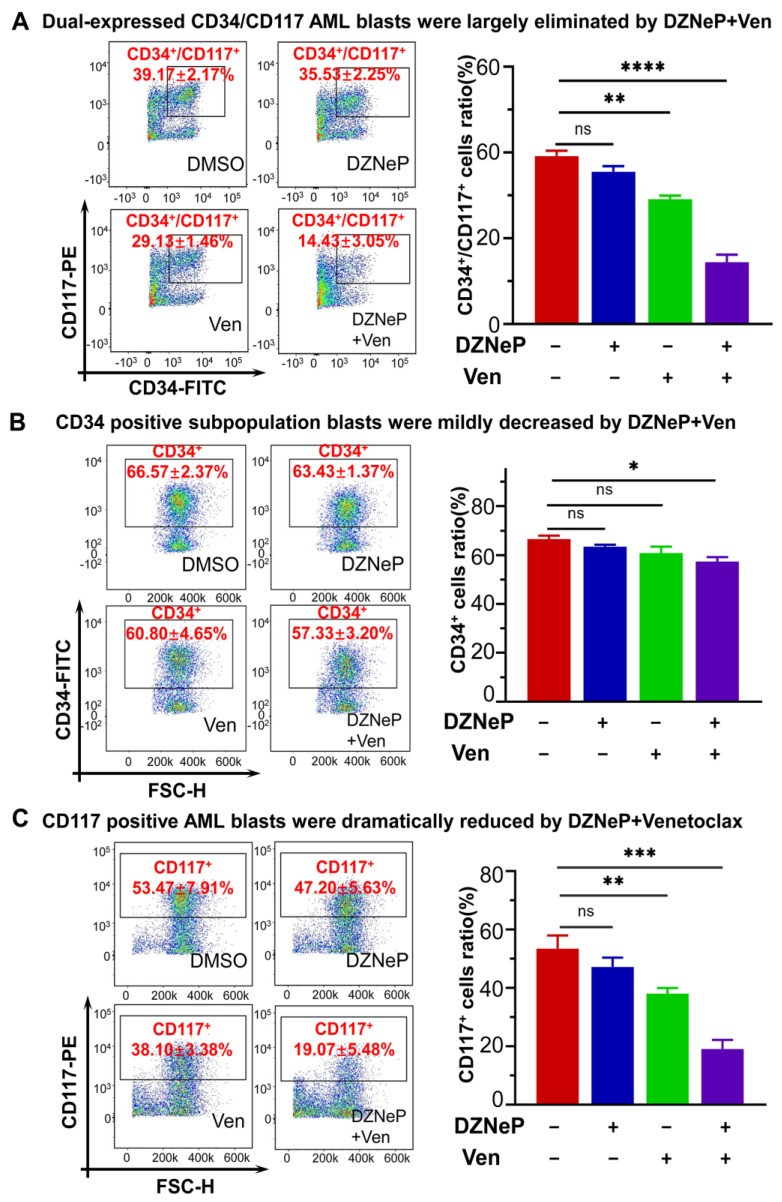
Effect of DZNeP + Ven on the cell growth arrest of c-KIT(CD117)-enriched primary AML blasts. (**A**–**C**) Effect of DZNeP + Ven on the cell growth arrest in dual CD34+CD17+ (**A**), CD34+ (**B**), and CD117+ (**C**) AML-M5b primary cells. Cells were treated with the indicated drugs for 48 hrs and analyzed by CCK-8 assay. Mean ± SD of triplicates is representative of independent experiments. ns, no significant. * *p* < 0.05, ** *p* < 0.01, *** *p* < 0.001, **** *p* < 0.0001.

Both c-KIT and CD34 are hematopoietic stem/progenitor markers; particularly, c-KIT is also the leukemia stem cell marker. Therefore, we examined the effect of the DZNeP + Ven combination on the primary cells expressing c-KIT and/or CD34. Results show that the dual CD34+CD117+ cells were significantly diminished after being treated by DZNeP + Ven for 48 h compared to the control or single-agent treatments (*p* < 0.0001) (Figure 6A). Cells with only CD34+ were slightly affected, but cells with only CD117+ were significantly reduced in the combination group compared to single-drug controls (Figure 6B,C). These data indicate that the DZNeP + Ven combination mainly suppresses the growth of the c-KIT-enriched cells, which suggestes the potential effect of the combination on the LSCs.

## 3. Discussion

The potent BCL-2 selective inhibitor, Ven, has been used for the therapy of AML with a good response in the initial treatment. However, the efficacy of the drug as a single agent has been underwhelming to date owing to the drug resistance, especially for monotherapy [35,36,37]. Feedback activation of parallel PI3K/AKT oncogenic pathways has been invoked as a major mechanism of drug resistance [38,39]. Numerous studies have suggested that co-targeted PI3K and BCL-2 signaling showed an enhancement of anti-leukemic activity [37,40,41,42,43]. In this study, we found that the EZH2 inhibitor, DZNeP, promotes the effect of the BCL-2 inhibitor, Ven, on cell growth arrest and apoptosis in AML cell lines and in primary leukemic cells from AML patients in a PIK3IP1-dependent manner. The mechanism model is summarized in Figure S2.

PIK3IP1 is an upstream molecule of the PI3K/AKT/mTOR pathway; it could directly bind to p110 of PI3K and downregulate the activation of the PI3K/AKT/mTOR pathway [44]. Here we found that PIK3IP1 is upregulated by DZNeP but downregulated by Ven. Re-activation of PI3K/AKT is the key reason for the resistance of BCL-2 inhibitor treatment in cancer [40,41,42,43,45]. Our data showed that PIK3IP1, the suppressor of PI3K/AKT/mTOR signaling, is significantly downregulated in AML cells and that PIK3IP is significantly elevated upon the combination of DZNeP with Ven. Our results not only revealed that PIK3IP1 downregulation may be the key reason responsible for the PI3K/AKT re-activation-mediated resistance of the BCL-2 inhibitor in cancer cells, but also highlighted the effect of targeting the EZH2/PIK3IP1 axis to suppress the PI3K/AKT/mTOR signaling anti-effect in AML.

CD34+/CD117+ cells have the potency for self-renewal and pluripotent differentiation, particularly, c-KIT (CD117), known as a stem cell factor (SCF) receptor, plays an essential role in stem cell maintenance and differentiation [46]. Normal hematopoietic cells express only a few c-KIT; while c-KIT is a leukemia stem cell marker, it is overexpressed to influence the malignant phenotype in AML cells [47]. Accumulated evidence has suggested that SCF signaling via c-KIT on hematopoietic stem/progenitor cells balances quiescence with activation and involves the PI3K/AKT signaling pathway [48,49,50]. Our data shows that a combination of DZNeP and Ven mainly suppresses c-KIT-enriched AML blasts. Our results indicate the effect of DZNeP on not only sensitizing the BCL-2 inhibitor and avoiding the re-activation of PI3K/AKT/mTOR signaling, but also on the inhibition of LSCs in AML.

We observed that EZH2 is highly expressed in AML patients, and the high expression is associated with poor outcomes (low CR rate, high refractory/relapse rate, and short OS) in the patients, but the EZH2-high expression was not observed to be associated with genomic defects. The epigenome-wide analysis revealed that EZH2 was highly expressed in embryonic stem cells and significantly downregulated in mesenchymal stem cells, and the EZH2 inhibitor, GSK126, induces embryonic stem cell differentiation into non-mesoderm and mesenchymal hepatocytes by inhibiting H3K27me3 [51]. The combination therapy of Ven with azacytidine or posaconazole induced deep AML remission with the eradication of LSCs and remodeling of the clonal hematopoiesis [52,53]. In the present study, we indeed observed that the combination of the EZH2 inhibitor, DZNeP, with Ven suppressed the c-KIT-enriched LSCs, which is consistent with these reports. We considered the epigenetic mechanisms that may be involved in the changes in the DZNeP-induced c-KIT expression in AML cells, although the detailed mechanisms need to be further explored in the future.

AML is classified into the M0-M7 subtypes based on the FAB classification. We observed the synergistic effect of the combination of DZNeP with Ven in primary cells from patients with the AML-M5b subtype and two available monocytic AML cell (U937 and MV4-11) lines. To examine if the combination prefers a subtype, we analyzed the association of the EZH2 expression with the clinical feature in our AML cohort and found that there are no significant differences in the EZH2 expression observed in the AML subtypes (Table S1). Similar data were achieved for the PI3KIP1 expression in our cohort (Table S1). These data reveal the therapeutic effect of the combination may apply to all AML subtypes, although it needs to be examined in primary cells from more patients with different subtypes.

Also, in this study, we observed the sensitivity difference in U937 and MV4-11 to BCL-2 inhibitors. U937 cells are highly insensitive to the BCL-2 inhibitor, with the IC50 values against Ven 1000-fold higher than the MV4-11cells. The reason for the difference needs to be further defined. However, MV4-11 cells, not U937 cells, have the MLL-AF4 fusion gene, which suggests that AML patients with the MLL-AF4 fusion gene may benefit from Ven monotherapy.

In summary, the combinatorial blockade of the EZH2 and BCL-2 shows a great synergistic anti-leukemia effect through upregulated PIK3IP1 and downregulated c-KIT. The preclinical data provided a rationale for the further development of the promising chemo-free combination of DZNeP with Ven for the treatment of AML patients ineligible for intensive chemotherapy, particularly in certain subtypes of poor-risk patients with high EZH2 and/or low PIK3IP1.

## 4. Methods and Materials

### 4.1. Clinical Samples and AML Primary Cells

The 88 patients with newly diagnosed AML and 70 healthy controls were recruited from 4 March 2016 to 30 January 2022, at Zhongda Hospital Southeast University (2016ZDKYSB062, 4 March 2016; 2017ZDSYLL067-P01, 10 August 2017; 2019ZDSYLL121-P01, 20 August 2019). Bone marrow aspirate samples were collected after the acquisition of written informed consent by the tenets of the Declaration of Helsinki and were approved by the Independent Ethics Committee for Clinical Research of Zhongda Hospital Southeast University. Bone marrow aspirate samples were collected, and mononuclear cells were separated using a lymphocytes separation medium (Ficoll, Shanghai, China), following the instructions, and were washed once with normal saline. The total RNA was isolated from the samples using TRIzol as described previously, and the complementary DNA was prepared for the detection of the target genes by RT-qPCR.

Newly diagnosed AML patients presenting with high leukocyte counts consented to undergo leukapheresis to collect primary cells. Mononuclear cells were enriched using Ficoll, and erythrocytes were lysed with a red blood cell lysis buffer (Biosharp, China). Primary AML cells were cultured in RPMI 1640 with 10% FBS and were subsequently used in various experiments.

The primary AML specimens used for culture in this study were isolated from the blast cells of a 15-year-old male with newly diagnosed AML-M5b. The immunophenotyping test report showed that the positively expressed cellular differentiation markers included CD117 (87.44%), CD34 (77.8%), CD45 (94.74%), CD13 (95.89%), CD64 (95.17%), CD33 (80.88%), CD38 (79.54%), CD4 (71.08%), CD11B (30.79%), and HLA-DR (6.31%). The negatively expressed CD markers included CD15, CD10, CD3, CD19, CD56, CD2, CD14, CD20, and CD7. No genomic defects were identified.

### 4.2. Cell Lines and Cell Culture

MV4-11 (CRL-9591), U937 (CRL-1593.2), and HEK-293T (CRL-3216) were purchased from the American Type Culture Collection (ATCC, Philadelphia, PA, USA). The MV4-11 cell line was cultured in IMDM supplemented with 10% fetal bovine serum (FBS). U937 was grown in RPMI 1640 medium supplied with 10% FBS. The medium was replaced every 2 to 3 days, and AML cell lines were passaged at a density of 2 × 10^6^/mL. HEK-293T cells were cultured in DMEM containing 10% FBS, and cells were subcultured when the fusion rate reached 80–90%. All cell lines were cultured at 37 °C with a 5% CO_2_ atmosphere. Logarithmically growing cells were prepared for all experiments described below.

### 4.3. Reagents

DZNeP (Cat.No S7120) and Ven (Cat.No S8048) were purchased from Selleck (Shanghai, China). For in vitro experiments, DZNeP and Ven were dissolved in anhydrous dimethyl sulfoxide (DMSO). IMDM was taken from HyClone Cytiva (Shanghai, China), and other mediums and FBS were obtained from Gibco (Beijing, China). The Cell Counting Kit-8 (CCK-8) was purchased from Dojindo Laboratories (Kumamoto, Japan). For flow cytometry antibodies, mouse anti-human MAbs and apoptosis antibodies were purchased from BD Biosciences (BD, USA), including anti-CD34-FITC, anti-CD117-PE, the FITC Annexin V apoptosis Detection Kit, APC Annexin V, and 7-AAD. The antibodies for the western blot were cleaved caspase-3 (9664, CST), PARP (9542, CST), BCL-2 (Ab32124, Abcam), BAX (Ab32503, Abcam), MCL1 (66026-1-Ig, Proteintech), PIK3IP1 (NBP1-69623, Novusbio), and GAPDH (Ab181602, Abcam). The PCR primers of EZH2, phosphoinositide-3-kinase-interacting protein1 (PIK3IP1), and GAPDH were synthesized by Sangon Biotech (Shanghai, China). The shRNA plasmid to PIK3IP1 was purchased from Corues Biotechnology (Nanjing, China). The ExFect transfection reagent was obtained from Vazyme (Nanjing, China).

### 4.4. Cell Proliferation Assay

The proliferation of cells was determined via CCK-8 according to the manufacturer’s instructions. Cells were plated in a 96-well plate at an initial density of 2 × 10^5^ per well in a 50 μL growth medium, and a 50 μL drug medium at seven serial diluted concentrations was added to the cell suspension. After 24 or 48 h of incubation, 10 µL of CCK-8 test solution per well was added to the 96 well plates. The plates were read after 2 to 4 h of incubation using 544 nm excitation and 590 nm emission wavelengths (ELx800, BioTek, Shoreline, WA, USA). Relative cell viability was normalized to DMSO-treated wells.

### 4.5. Transcriptome RNA Sequence

U937 AML cells were treated with 2 μM of DZNeP, 7.5 μM of Ven, or vehicle for 48 h, respectively. The total RNA was extracted using a TRIzol reagent (Takara Bio, Beijing, China). The RNA-seq libraries were constructed using the TruSeq RNA Sample Preparation Kit following the manufacturer’s instructions. In addition, 150 bp paired-end sequencing was supported by the Illumina HiSeq platform (Novegene, Nanjing, China).

High-throughput transcriptome sequencing technology was performed for the mRNA expression profiles following DZNeP, Ven, or vehicle treatment upon U937 AML cells. Differential expression genes (DEGs) between the two groups were identified using the DEseq2 R package, and a list of genes was considered statistically significant with |log2 Fold Change| > 1.0 and FDR < 0.05. For the visualization of the RNA-seq datasets, volcano plots were created showing log2 of the relative fold change in the genes in the compared groups, and corresponding FDR values were transformed into -log10 values. In addition, the cluster profiler R package was used to assess the statistical enrichment of DEGs in KEGG (Kyoto Encyclopedia of Genes and Genomes) pathways.

### 4.6. Bioinformatics Analysis of Public Databases

The R2: Genomics Analysis and Visualization Platform is a freely accessible web-based genomic analysis and visualization application that can analyze a large collection of public data [54]. The R2 was used to compare the expression of EZH2 and PIK3IP1 between the AML patients and normal samples. Gene expression data for these AML patients were deposited online at NCBI GEO with accession numbers GSE37642 [55], GSE4608 [56], GSE6891 [57], GSE10358 [58], GSE12417 [59], GSE111678 [60], GSE17855 [61], GSE21261 [62], GSE22845 [63], GSE14468 [64], and GSE15434 [65]. The datasets for the normal controls are available through GEO under accession numbers GSE46510 [66], GSE7158 [67], and GSE28491 [68]. The prognostic value of EZH2 and PIK3IP1 in AML patients was determined by analyzing the database of 422 AML patients from GSE37642 [55]. The Kaplan–Meier overall survival analysis was also conducted by the R2 platform.

### 4.7. Real-Time Quantitative PCR Assay

RT-qPCR was used to detect the mRNA expression of interested genes among different treatment groups. The total RNA was extracted by TRIzol, phase-separated with chloroform, precipitated with isopropyl alcohol, washed with 75% ethanol, and redissolved in RNAase-free water. Reverse transcription was performed with 0.5 μg of RNA using the PrimeScript™ RT Master Mix reverse transcriptase kit (Takara, Japan) following the manufacturer’s instructions. The ABI StepOnePlus RT PCR System (Applied Biosystems, Foster City, CA, USA) was used to perform the amplification reaction using TB Green^®^ Premix Ex Taq™ (Tli RNaseH Plus) (Takara, Japan) according to the manufacturer’s instructions. The relative abundance of GAPDH mRNA was used to normalize the levels of the mRNAs of the target, and each sample was repeated three times.

With the same approaches, the mRNA levels of EZH2 and PIK3IP1 were examined with qPCR analysis of the patients’ samples. Target gene expression levels were calculated by 2^(−∆Ct)^. The patients were classified into two groups: “EZH2-high” and “EZH2-low” based on their relative gene expression levels, i.e., “EZH2-high” includes the patients whose mRNA levels were within the highest 25%, while “EZH2-low” was for those in the lowest 75% in the cohort. The patients were also classified into two groups based on their PIK3IP1 mRNA levels; the “PIK3IP1-High” was for those within the highest 75%, while the “PIK3Ip1-low” was for those in the lowest 25% in the cohort.

### 4.8. Western Blot

Protein expression levels were measured by western blot. MV4-11 or U937 AML cells were treated with control, DZNeP, Ven, or DZNeP + Ven for 48 h and lysed with a RIPA lysis buffer (KenGen Biotech, Nanjing, China); then, the concentration of the total protein was quantified using a BCA Protein Assay kit (Vazyme, Nanjing, China) following the instructions. Cell lysates were mixed with a 5 × SDS loading buffer and heated at 100 °C for 10 min. Next, protein samples were loaded with 7.5–10% SDS-PAGE gels along with a pre-stained protein marker and were isolated via constant voltage electrophoresis. Proteins were then wet transferred onto a polyvinylidene difluoride membrane (PVDF; USA). The membrane was blocked with 5% nonfat milk for 1 h at room temperature and incubated with primary antibodies overnight at 4 °C, followed by incubation with horseradish peroxidase (HRP)-conjugated secondary antibodies for 1 h at room temperature. Later, the bands were visualized with an enhanced chemiluminescence assay (ECL) kit and photographed using an Amersham Imager 600 (GE Healthcare Life Sciences, Chicago, IL, USA). GAPDH was used as an internal control.

### 4.9. Flow Cytometry Assay

Apoptosis was estimated by flow cytometry measurements. Briefly, the cells differently treated were collected by centrifugation for 5 min at 300× *g* at 4 °C, washed in PBS twice, and then cells were suspended in 100 μL of 1xAnnexin V binding buffer. Subsequently, the MV4-11 and U937 cells were added with 5 μL of Annexin V-FITC and 5 μL of propidium iodide (PI), while the transfected cells and AML blasts in the CD34+/CD117+ cells were dual-stained with 5 μL of Annexin V-APC and 5 μL of 7-Amino-Actinomycin D (7-AAD), followed by incubation for 15 min at room temperature in the dark, according to the manufacturer’s protocol. A total of 400 µL of 1X binding buffer was added to each sample before being analyzed by flow cytometry (FACScan, BD Biosciences, San Diego, CA, USA).

To quantitate the expression of cell surface biomarkers, the membrane surface expression of CD34 and CD117 from different treatment groups in blasts from the patient with AML-M5b by apheresis was studied using flow cytometry. Samples were stained simultaneously with anti-human CD34 and CD117 antibodies following incubation for 15 min at room temperature in the dark. Unbound antibodies were washed away and measured via flow cytometry. Data analysis was performed using FlowJo v10 software (FlowJo, LLC, Ashland, OR, USA).

### 4.10. PIK3IP1 shRNA Knockdown

PIK3IP1-specific shRNA was obtained from Corues Biotechnology. For lentivirus production, HEK-293T cells were transfected with PIK3IP1 shRNA, psPAX2, and pMD2.G using the ExFect transfection reagent T101 according to the lipofectamine manual (Vazyme, Nanjing, China). MV4-11 or U937 cells were infected with a viral supernatant containing 8 μg/mL of polybrene for 72 h. The pLV3ltr-ZsGreen-puro-U6 vector was used as the vehicle control. Cells were selected with puromycin for 7 days followed by puromycin maintenance. The infection efficiency was assessed by western blot and RT-PCR.

### 4.11. Statistical Analysis

Statistical analysis was performed using IBM SPSS statistical software v26.0. Data graphics were conducted using Prism software v9.0 (GraphPad Software, San Diego, CA, USA). Experiments were performed in independent triplication as indicated. The quantitative data were presented as the mean ± SD, and the qualitative data were expressed by the number of cases and percentages. The comparison of measurement data was performed by Student’s *t*-test between the two groups, while multiple-group comparisons were performed using one-way ANOVA followed by an LSD post-hoc test. To determine the correlations between the mRNA levels of EZH2 and PIK3IP1, simple linear regression and Pearson’s correlation analysis were performed. The R coefficient and the relative *p*-values were evaluated, and a good correlation between variables was considered for R  ≥  0.35 and *p* < 0.05.

Qualitative data are presented as absolute counts and relative frequencies. Quantitative data are presented as mean ± standard deviation. For age, we used the Student’s t-test; for WBC, Hb, PLT, and BM blasts, we used the Mann–Whitney test; the chi-squared test was used for the comparison of the categorized data between the two groups. The overall survival (OS) was defined as the time from the diagnosed date until death from any cause. Survival probabilities with two-sided 95% CIs were estimated using the Kaplan–Meier method, and differences were tested for statistical significance with the (two-sided) log-rank test. A two-sided *p* < 0.05 was considered statistically significant.

## Data Availability

The patient datasets for the current study are not publicly accessible by local health research ethics protocols; however, they may be available from the corresponding author.

## Supplementary Materials

The following supporting information can be downloaded at: https://www.mdpi.com/article/10.3390/ijms231911393/s1.
