# Supplementary material for: Targeting EZH2 Promotes Chemosensitivity of BCL-2 Inhibitor through Suppressing PI3K and c-KIT Signaling in Acute Myeloid Leukemia
Source: Int J Mol Sci. 2022 Sep 27;23(19):11393. doi: 10.3390/ijms231911393 (PMC9569949; doi:10.3390/ijms231911393)
Supplement: Supplementary file 1 [file ijms-23-11393-s001.zip › ijms-1896271-supplementary.pdf]

Supplemental Table and Figures

Table S1 Correlation of EZH2 and PIK3IP1 expression with clinical features in AML cohort

Characteristics	EZH2 Expression (N=88)			PIK3IP1 Expression (N=88)		
	High(n=22)	Low(n=66)	p Value	High(n=66)	Low(n=22)	p Value
**Gender, n (%)**						
Male	11 (50.0%)	28 (42.4%)	0.536	30 (45.5%)	9 (40.9%)	0.710
**Age, median (range), y**	66 (24-88)	55 (18-82)	**0.033**	61 (18-88)	58 (24-88)	0.855
**FAB subtype of AML, n (%)**			0.258			0.791
M0	1 (4.5%)	1 (1.5%)		1 (1.5%)	1 (4.5%)	
M1	2 (9.1%)	5 (7.6%)		5 (7.6%)	2 (9.1%)	
M2	13 (59.1%)	28 (42.4%)		33 (50.0%)	8 (36.4%)	
M3	0 (0.0%)	4 (6.1%)		2 (3.0%)	2 (9.1%)	
M4	1 (4.5%)	6 (9.1%)		5 (7.6%)	2 (9.1%)	
M5	4 (18.2%)	22 (33.3%)		19 (28.8%)	7 (31.8%)	
M7	1 (4.5%)	0 (0.0%)		1 (1.5%)	0 (0.0%)	
**WBC ($x10^9$/L), median(range)**	7.5 (0.34-58.54)	13 (0.37-201.9)	0.088	8 (0.34-201.9)	15 (0.37-179.7)	0.099
**Hb (g/L), median(range)**	71 (37-118)	77.5 (31-123)	0.700	75 (31-120)	82.5 (36-123)	0.182
**PLT ($x10^9$/L), median(range)**	22.5 (5-265)	42.5 (3-252)	**0.047**	38.5 (3-265)	39 (14-252)	0.795
**BM blasts (%), median (range)**	51 (4-96)	60.4 (17-97.6)	0.221	58.6 (17-96)	56 (4.80-97.6)	0.658
**Cytogenetics, n (%)**						
Normal	9 (47.4%)	36 (58.1%)	0.444	34 (45.0%)	11 (55.0%)	1.000
Complex Karyotype	5 (26.3%)	7 (11.5%)	0.114	10 (16.7%)	2 (10.0%)	0.470
7q-/-7	3 (15.8%)	1 (1.6%)	**0.013**	2 (3.3%)	2 (10.0%)	0.236
**Gene mutations, n (%)**						
TP53	3 (13.6%)	3 (4.5%)	0.143	1 (1.5%)	5 (22.7%)	**0.001**
ASXL1	2 (9.1%)	2 (3.0%)	0.237	3 (4.5%)	1 (4.5%)	1.000
DNMT3A	2 (9.1%)	15 (22.7%)	0.161	4 (6.1%)	13 (59.1%)	**<0.001**
NPM1	1 (4.5%)	15(22.7%)	0.056	7 (10.6%)	9 (40.9%)	**0.001**
KRAS	0 (0.0%)	2 (3.0%)	0.409	1 (1.5%)	1 (4.5%)	0.440
NRAS	1 (4.5%)	4 (6.1%)	0.790	3 (4.5%)	2 (9.1%)	0.425
FLT3-ITD	0 (0.0%)	8 (12.1%)	0.087	5 (7.6%)	2 (9.1%)	0.820
**Complete response, n (%)**	13 (59.1%)	57 (86.4%)	**0.006**	54 (81.8%)	16 (72.7%)	0.360
**Refractory/Relapse, n (%)**	9 (45.0%)	14 (21.2%)	**0.035**	15 (23.1%)	8 (38.1%)	0.176
**Median OS, months (95% CI)**	12.0 (1.71-22.29)	NR	**0.007**	30.0	20.0 (7.05-32.95)	0.500

WBC, white blood cells; Hb, hemoglobin; PLT, Platelet count; BM, bone marrow; Complex karyotype, defined as having 3 or more chromosomal abnormalities in the absence of one of the WHO–designated recurrent genetic abnormalities; OS, overall survival; SE, Standard error; CI, Confidence Interval; NR, not reached.

Table S2 Clinical Features of the patients in the AML cohort

Patient ID	FAB subtype	Gender	Age	WBC(10^9/L)	Hb(g/L)	PLT(10^9/L)	BM Blasts	Molecular features	Cytogenetics	Treatment response	RFS(months)	Refractory/Relapse(yes/no)	OS(months)	Survival at last follow-up(alive/died)
AML_1	M2	Female	88	6.13	71	74	34.00%	negative	Normal	NR		no	5	died
AML_2	M2	Female	70	57.06	80	21	34.80%	negative	t（9,22）	NR		yes	12	died
AML_3	M4	Female	35	30.67	123	62	68.40%	CBFβ/MYH11	46,XX inv(16)p13 q22）/(20)	CR	29	no	30	alive
AML_4	M2	Female	62	2.73	80	46	63.20%	AML1/ETO	46,XX,t(8;21)(q22;q22)[1]/46,I dem,del(9)(q22)[1]/45,I dem,-X,del(9)(q22)[3]/46,XX[15]	CR	53	no	54	alive
AML_5	M2	Female	49	20.47	76	33	40.40%	FLT3-ITD, NPM1	Normal	NR	38	no	42	alive
AML_6	M5	Female	55	6.58	65	102	31.20%	NPM1，CEBPA	Normal	CR	6	no	5	alive
AML_7	M5	Female	54	44.98	121	116	61.60%	NPM1、DNMT3A	Normal	CR	60	no	61	alive
AML_8	M5	Female	81	44.24	102	79	60%	HOX11 rearrangement, NPM1、DNMT3A	Normal	NR	10	yes	16	died
AML_9	M4	Female	56	47.7	65	25	53.20%	FLT3-ITD	Normal	CR	29	yes	36	alive
AML_10	M1	Male	49	179.7	99	63	97.60%	FLT3-ITD, NPM1	Normal	PR	11	no	15	died
AML_11	M5	Female	61	58.54	100	22	59.20%	negative	Normal	CR	1	no	1	alive
AML_12	M3	Female	45	0.37	63	20	61.20%	PML/RARα	t(15,17）	CR		no	52	alive
AML_13	M2	Male	68	3.92	86	265	31.20%	IDH2	46,XY,del(7)(q31)[12]/46,XY[5]	PR	2	no	6	alive
AML_14	M2	Male	48	18.24	87	18	46%	HOX11/EVI1, NPM1	Normal	CR	21	yes	32	alive
AML_15	M2	Female	41	122.12	49	18	62.40%	FLT3-ITD	46,XX,del(20)(q13)[20]	NR		yes	2	alive
AML_16	M5	Female	48	32.81	109	37	85.60%	CBFβ/MYH11	46，XX，？add(7)(q11.2), inv(16)(p13q22)[20]	CR	46	no	47	alive
AML_17	M5	Female	82	11.62	107	10	65.60%	CEBPA	Normal	PR	1	no	1	alive
AML_18	M2	Male	75	201.9	96	36	89%	negative	Normal	PR		yes	2	died
AML_19	M5b	Female	73	108.15	95	42	65.60%	FLT3-ITD、NPM1	Normal	NR	1	yes	1	died
AML_20	M0	Male	55	25.66	82	31	76%	RUNX1	Normal	PR	2	no	2	alive
AML_21	M2	Female	88	8.47	81	14	24%	negative	Normal	PR	1	no	3	died
AML_22	M2	Male	64	2.99	83	63	26%	negative	47，XY，+1, der(1;19)(q10;q10),+19[19]/46,XY[1]	NR	7	no	8	died
AML_23	M4	Female	44	132.28	82	37	54.80%	FLT3-ITD	Normal	CR	1	no	2	alive
AML_24	M3	Female	25	0.8	74	93	55.20%	PML-RARα	47，XX，+8，t(15；17)(q22；q21)[14]/46,XX[6]	CR		no	27	alive
AML_25	M3	Female	50	52.67	71	82	93.30%	PML-RARα	t(15,17)	CR	17	no	18	alive
AML_26	M5	Male	75	39.54	57	39	36.40%	SRSF2	46,XY,del(20)(q11)[11]/47,idem,+8[9]	CR	2	no	2	died
AML_27	M5	Female	69	52.02	100	159	65.60%	DNMT3A, TET2	Normal	CR	11	no	12	died
AML_28	M1	Male	56	52.16	72	23	90.40%	negative	46，XY，add（21）（p11）[6]/46,XY[14]	CR	18	yes	16	died
AML_29	M4	Male	25	47.48	72	50	49.20%	FLT3-TKD, NRAS, CBFB/MYH11	46,XY,inv(16)(p13q22)[20]	CR	37	no	38	alive
AML_30	M5	Male	67	0.86	56	26	33.20%	ASXL1，BCOR，DNMT3A, IDH，RUNX1	Normal	NR	1	no	2	alive

Table S2 Clinical Features of the patients in the AML cohort (continue1)

Patient ID	FAB subtype	Gender	Age	WBC(10^9/L)	Hb(g/L)	PLT(10^9/L)	BM Blasts	Molecular features	Cytogenetics	Treatment response	RFS(months)	Refractory/Relapse(yes/no)	OS(months)	Survival at last follow-up(alive/died)
AML_31	M0	Female	60	35.48	53	15	48.80%	CBFβ/MYH11，NRAS	t(8,10); inv(16)	CR	4	no	5	alive
AML_32	M2	Male	67	2.35	99	51	62.80%	TET2	Normal	CR	25	yes	30	died
AML_33	M2	Male	42	154.9	82	33	72.80%	FLT3, CBFB/MYH11	46,XY,inv(16)(p13q22)[3]/47,idem,+22[15]/46,XY[2]	CR	13	no	14	died
AML_34	M2	Female	55	7.23	83	62	54.80%	TET2, STAG2, CEBPA	Normal	CR	38	no	39	alive
AML_35	M2	Male	78	0.34	69	20	75.60%	RUNX1, SF3B1	No mitotic phase	NR			1	died
AML_36	M2	Female	62	1.8	118	79	30.88%	WT1 high expression	Normal	CR	17	yes	26	died
AML_37	M2	Male	24	4.41	65	5	77.60%	AML1/ETO	No mitotic phase	CR	2	no	3	alive
AML_38	M2	Female	75	15.17	90	23	22.60%	AML1/ETO	45，X,-X,t（8；21）（q22；q22）[20]	CR	14	yes	92	alive
AML_39	M2	Female	40	13.6	44	17	70.80%	GATA2	No mitotic phase	CR	1	yes	30	died
AML_40	M2	Female	66	3.29	61	19	27.50%	negative	Normal	CR	2	no	3	died
AML_41	M2	Male	44	33.13	68	15	75.60%	KIT, AML1/ETO	Normal	CR	5	no	6	alive
AML_42	M2	Female	35	10.37	82	24	32.80%	AML1/ETO	46，XX，t(8；21)（q22；q22）[10]	CR	8	yes	15	died
AML_43	M2	Male	67	12.43	70	23	53.20%	TET2	Normal	PR		yes	13	died
AML_44	M2	Female	24	26.66	36	23	55.20%	WT1 high expression	46,XX,?del(9)(q22)[9]/46,XX[11]	CR	33	no	34	alive
AML_45	M3	Female	54	0.87	67	85	69.5%	PML/RARα	t (15,17)	CR	17	no	18	alive
AML_46	M5b	Female	53	69.32	53	30	46.80%	TET2，WT1，KRAS	Normal	CR	18	no	20	died
AML_47	M2	Female	50	1.08	89	45	63.50%	IDH2, CEBPA	No mitotic phase	CR	14	yes	34	alive
AML_48	M2	Female	48	14.43	31	3	60.80%	AML1/ETO, KIT	46,XX,inv(8)(p23q21),t(8;21)(q22;q22)[11]/47,idem,+4[4]	CR	13	no	14	alive
AML_49	M4	Female	43	62.12	81	43	75.50%	CBFβ/MYH11，KIT, FLT3	47,XX,inv(16)(p13q21),+22[20]	CR	18	no	19	alive
AML_50	M5	Female	34	0.81	64	25	87.60%	U2AF1, SETBP1	46,X,del(X)(q24),t(9;11)(p22;q23),?add(17)(q25)	NR	22	no	27	alive
AML_51	M2	Female	48	2.19	89	78	17%	IDH1，TET2	No mitotic phase	CR	19	no	20	alive
AML_52	M2	Male	43	1.6	62	43	23%	RUNX1, U2AF1, BCOR	Normal	CR	8	no	15	died
AML_53	M2	Female	21	6.72	68	18	44%	GATA2, CEBPA	Normal	CR	18	no	19	alive
AML_54	M5	Male	79	4.12	78	54	46.40%	TET2, DNMT3A	Normal	CR	4	no	6	died
AML_55	M2	Male	72	2.37	72	86	63.20%	TET2	Normal	CR	15	no	18	died
AML_56	M1	Female	75	47.99	71	53	54.40%	DNMT3A, TET2	Normal	NR	5	yes	5	died
AML_57	M7	Male	65	5.4	64	70	34.80%	TP53	Complex Karyotype	PR	-	yes	1	died
AML_58	M5b	Male	54	36.11	120	62	52%	KRAS	Normal	CR	16	no	17	alive
AML_59	M5	Female	66	1.39	66	24	58%	DNMT3A, IKZF1，IDH2，BCOR	Normal	CR	5	no	6	alive
AML_60	M2	Male	78	4.21	89	15	64.80%	ASXL1, IDH2, RUNX1, PHF6	Normal	PR		yes	3	died

Table S2 Clinical Features of the patients in the AML cohort (continue2)

Patient ID	FAB subtype	Gender	Age	WBC(10^9/L)	Hb(g/L)	PLT(10^9/L)	BM Blasts	Molecular features	Cytogenetics	Treatment response	RFS(months)	Refractory/Relapse(yes/no)	OS(months)	Survival at last follow-up(alive/died)
AML_61	M2	Female	75	11.43	95	43	77.00%	TP53， IDH2	43~45,X,-X,idic(11)(p11),-14,-15,-16,-17,-18,+4~6mar,inc[cp6]/46,XX[5]；FISH:7q-/-7：3%,+8：9%,17p-：94.5%， -17：18.7%,	NR				
AML_62	M4	Male	48	6.63	37	11	23.50%	ZRSR2	46， XY,inv（3）（q21q26)[6]/46,XY[4]	PR	9	yes	15	alive
AML_63	M2	Male	59	7.98	54	21	67.20%	KIT， ASXL1， AML1/ETO	46， XY， t（8；21）（q22；q22）[20]	CR	2	no	4	died
AML_64	M1	Male	47	23.36	85	67	96%	IDH1， FLT3， TP53	Normal	CR	2	no	3	alive
AML_65	M5	Female	64	1.22	61	20	40.00%	TP53， PTPN11， ASXL2	46, XXdel(l)(p11p12),del(5)(q13q33)+8,add(9)(q34),+11,der(17;20)(q10;p10),-18[20]	NR	1	yes	6	alive
AML_66	M5a	Female	70	25.98	79	88	85.20%	PTPN11， MLL/AF9	47， XX,+8[20]	CR	4	no	24	alive
AML_67	M4	Male	41	9.59	66	36	53.60%	CSF3R， WT1， CBFβ/MYH11	Normal	CR	23	no	24	alive
AML_68	M2	Male	48	10.6	77	23	63.20%	DNMT3A	Normal	CR	2	no	5	died
AML_69	M5	Male	57	4.8	75	67	24.40%	TP53	43-48， XY,+X,psu dic(5;3)(q35;p21),-7,add(11)(q13),-12,-13,add(17)(p13),+2~3mar[20]	CR	6	no	7	died
AML_70	M1	Female	70	7.68	97	12	89%	WT1	Normal	CR	15	no	17	alive
AML_71	M5	Female	18	0.4	36	25	54.40%	negative	No mitotic phase	CR	26	no	28	alive
AML_72	M2	Male	73	0.69	42	29	34.80%	ETV6	46,XY[20]， 17p- 6.5%	CR	21	no	22	alive
AML_73	M5	Male	78	73.87	91	90	49.60%	DNMT3A， TET2， NPM1	Normal	CR	3	no	6	died
AML_74	M2	Male	74	8.28	119	66	53.20%	DNMT3A， CEBPA	Normal	CR	11	no	12	alive
AML_75	M2	Male	71	5.61	84	105	56.80%	DNMT3A， TET2， NPM1， FLT3-ITD	No mitotic phase	CR	7	no	8	died
AML_76	M1	Male	73	7.71	68	50	92.80%	TET2， DNMT3A， NPM1	Normal	CR	16	no	17	alive
AML_77	M2b	Female	26	33.55	56	37	48.80%	AML1/ETO， PTEN， ASXL1， CSF3R	46,XX， t（8；21）（q22；q22)[19]/46,XX[1], RUNX1/RUNX1T1+:99%	CR	2	no	3	alive
AML_78	M5	Male	70	10.2	101	252	44%	DNMT3A， NPM1， IDH1， NRAS	Normal	CR	1	no	2	died
AML_79	M2	Female	69	7.79	55	121	20.40%	RAD21， AML1/ETO	45,X,t（8；21）（q22；q22）[17]/46,XX[3]	CR	6	no	7	alive
AML_80	M1	Female	75	5.12	75	21	81.00%	DNMT3A, TET2CEBPA:NM_004364.4:exon1:c.1042_1043dupCT:p.Ser348ArgfsTer75 38.78% 移码突变；CEBPA: NM_004364.4:exon1:c.484C>T:p.Q162X 38.85% 无义突变；WT1/ABL1 0.250%；	46, XX,del(11)(q14)[12]/47,idem,+?8[2]/46,XX[2]	CR	1	no	2	alive
AML_81	M5b	Male	64	20.66	58	28	27.60%	SF3B1， FLT3-ITD， RUNX1	Normal	CR	17	no	18	alive
AML_82	M2	Female	64	1.71	92	109	62.00%	NPM1， IDH1， DNMT3A	Normal	CR	2	no	3	alive
AML_83	M5b	Female	73	101.82	109	38	76.31%	TET2， GATA1， NRAS， NPM1	46， XX， +1， psu dic(4;1)(p16;p11)[9]/46,XX[1]	NR		yes	2	alive
AML_84	M2	Male	63	1.54	62	99	76.00%	TET2， DNMT3A， NPM1	Normal	CR	10	no	11	alive
AML_85	M2	Male	71	6.38	60	54	48.00%	TP53	42,X,-Y,t(2;2)(p24;p13),del(3)(q26.2),inv(4)(p16q12),del(5)(q23),+11,der(11;14)(q10;q10),-17,-18,del(20)(q12),-21[20]	CR	4	no	6	died
AML_86	M5b	Male	59	95.6	78	68	80.40%	DNMT3A， NPM1， IDH2， NRAS	Normal	CR	9	no	10	alive
AML_87	M5b	Female	53	11.88	97	85	77.00%	CBL， NPM1	No mitotic phase	CR	3	yes	8	alive
AML_88	M5a	Male	69	9.25	116	140	96.00%	WT1 high expression	47~48， XY， del(1)(q32), del(5)(q15q31),-7,add(9)(p13),-12,+19,-22,+2~3mar[cp4]/46,XY ,del(5)(q15q31),-7,-19,-22,+mar1,+mar2,+mar3[3]/46XY [13]。5q-:65%,7q-/-7 94%	PR		yes	4	died

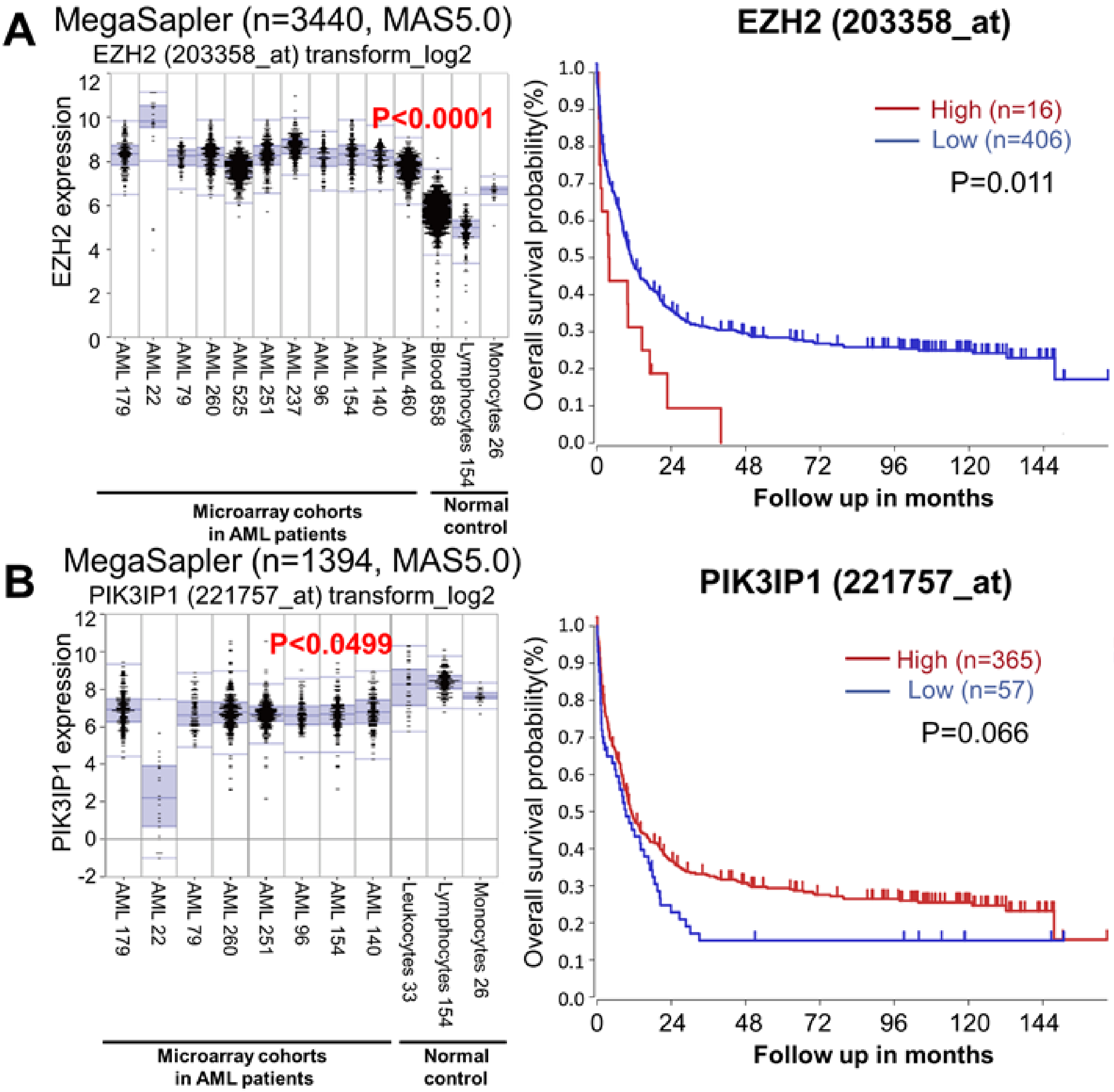

Figure S1. Expression of EZH2 or PIK3IP1 in blood or bone marrow samples of AML patients Versus normal controls from MegaSapler dataset and their association with survival. (A, B) The mRNA level of EZH2 is upregulated (A, left panel) while PIK3IP1 is downregulated (B, left panel) in AML cohorts compared to that of normal control. Association of EZH2^{lower} or PIK3IP1^{higher} with a longer OS compared to EZH2^{Higher} or PIK3IP1^{Lower} (A & B, right panel).

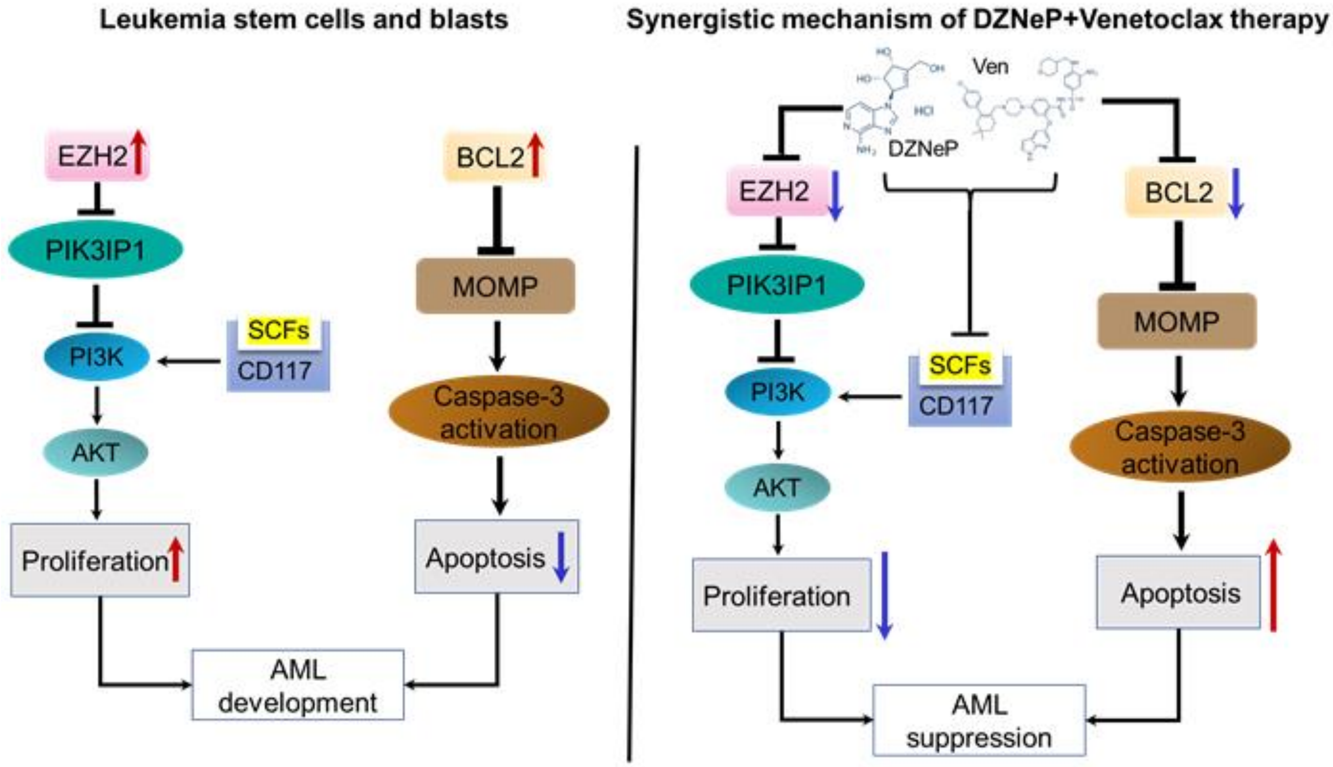

Figure S2. Mechanism model for the synergistic anti-leukemic effect of DZNeP+Ven in AML. In leukemia cells, highly expressed EZH2 and BCL2 promote the proliferation of AML cells and suppress apoptosis through activation of PI3K/AKT signaling. CD117（c-KIT）is also activated through PI3K/AKT signaling. DZNeP treatment increases the expression of PIK3IP1, the PI3K/AKT signaling suppressor, which causes a significant proliferation arrest; and Ven induces apoptosis through promoting MOMP. Combination treatment of DZNeP+Ven shows the synergistic effects of proliferation arrest and apoptosis through enhancing expression of PIK3IP1 and decreasing c-KIT in AML. (Abbreviation: SCFs, stem cell factors; MOMP, mitochondrial outer membrane permeabilization)
